# Capturing what matters: Patient‐reported LGI1‐ANTibody encephalitis outcome RatiNg scale (LANTERN)

**DOI:** 10.1002/acn3.70006

**Published:** 2025-02-25

**Authors:** Mark J Kelly, Barbara Wagner, Bryan Ceronie, Christine Strippel, Ann Yee Lin, Adam Handel, John Soltys, Sophie Binks, Philip A Powell, Sarosh R Irani

**Affiliations:** ^1^ Oxford Autoimmune Neurology Group, Nuffield Department of Clinical Neurosciences University of Oxford Oxford UK; ^2^ Department of Physiology and Medical Physics Royal College of Surgeons in Ireland Dublin Ireland; ^3^ Department of Neurology John Radcliffe Hospital Oxford UK; ^4^ Department of Neurology Kantonsspital Aarau Aarau Switzerland; ^5^ Department of Neurosciences Mayo Clinic Jacksonville Florida USA; ^6^ Department of Neurology University of South Alabama Mobile Alabama USA; ^7^ Sheffield Centre for Health and Related Research (SCHARR) University of Sheffield Sheffield UK; ^8^ Department of Neurology Mayo Clinic Jacksonville Florida USA

## Abstract

**Background:**

LGI1‐antibody encephalitis (LGI1‐Ab‐E) is a common form of autoimmune encephalitis where most patients demonstrate ‘good’ clinician‐rated outcomes. However, more targeted questionnaires reveal numerous debilitating symptoms for many years. To better quantify these persistent features, we designed the LGI1‐Antibody Encephalitis Rating (LANTERN) scale, a quantified, disease‐specific patient‐reported outcome measure (PROM), adhering to FDA guidelines.

**Methods:**

A participant‐driven mixed‐methods approach to develop a clinically valid questionnaire over three stages: (1) Item generation through semi‐structured interviews; (2) Repeated cognitive debriefing rounds to advance comprehensibility, relevance and comprehensiveness; (3) Psychometric survey to condense the most sensitive and valid questions. Analyses incorporated sensitivity testing with multiple internal and external validations.

**Results:**

From 73 items across six domains (Stage 1; *n* = 18), a questionnaire assessing the frequency and severity of 43 symptoms (80 questions), plus nine activities of daily living (ADL), was developed through cognitive debriefing (Stage 2; *n* = 15). This 89‐question survey was completed (Stage 3; *n* = 66 patients and 32 relatives) and distilled, using exploratory factor analyses, to a three‐factor symptom‐burden questionnaire comprising 41 questions (19 symptoms and 6 ADL), separated into physical, cognitive/behavioural and ADL domains. These factors demonstrated strong internal reliability (Cronbach alpha: 0.85–0.91), correlations with relative‐completed questionnaires (*R* = 0.73–0.85; *p* < 0.001), good‐to‐excellent intraclass re‐testing correlations (0.81–0.98; *n* = 19) and strong associations with numerous predefined external measures.

**Discussion:**

LANTERN represents a PROM for LGI1‐Ab‐E, with initial content, structural and construct validity and test–retest reliability. It can be used as a reliable, tailored, efficient and sensitive method to establish symptom burden in people with LGI1‐Ab‐E, both in clinical practice and trials.

## Introduction

Autoimmune encephalitis (AE) is an umbrella term used to describe a complex syndrome characterised by seizures, cognitive impairment, psychiatric features, movement disorders and autonomic disturbances. AE is more frequent than infectious encephalitis, and many AE cases are caused by autoantibodies targeting neuronal antigens. Of these, LGI1‐antibody encephalitis (LGI1‐Ab‐E) is one of the most common.^1^ LGI1‐Ab‐E is characterised by subacute onset of cognitive impairment, behavioural changes and frequent focal seizures, including pathognomonic faciobrachial dystonic seizures (FBDS) in 50% which can occur at sometimes hundreds per day.^2^ The median age of onset is in the seventh decade, with 2:1 male predominance.^3^ Antiseizure medications (ASM) have limited effects, whereas >90% of seizures resolve with immunotherapies,^4^ particularly corticosteroids.^5^ Despite seizure resolution in most cases, residual cognitive impairments are common and recent studies using targeted questionnaires have additionally identified persistent features including affective disturbances and fatigue.^6^, ^7^

The current standard measures of outcome and disease severity in AE are the modified Rankin Scale (mRS)^8^ and the Clinical Assessment Scale in Autoimmune Encephalitis (CASE).^9^ The mRS was originally developed to measure outcomes following stroke: hence, it is strongly influenced by mobility and independence with little focus on neuropsychiatric symptoms or quality‐of‐life (QoL). Yet, in AE, many who demonstrate ‘favourable’ outcomes (mRS ≤2) still have measurable, sometimes marked, deficits in cognition, mood and fatigue.^6^, ^7^ The CASE is considered more disease‐specific but was developed to capture all forms of AE at the illness nadir.^10^ Consequently, it includes clinical parameters not typically associated with LGI1‐Ab‐E, including dyskinesias, brainstem dysfunction, level of consciousness and limb weakness. Furthermore, both the mRS and CASE are clinician‐, not patient‐, rated and hence fail to capture this paramount ‘survivor’ perspective. Also, existing scales used in AE fail to capture independent effects of each symptom's burden and its actual frequency, potentially important differences.^6^, ^7^, ^11^, ^12^

Hence, there is an urgent clinical and research requirement for a robust disease‐specific patient‐reported outcome measure (PROM) which adequately quantifies features of LGI1‐Ab‐E considered to be most impactful to patients and/or their relatives in the recovery phase. This would help more accurately assess treatment efficacy and outcomes in LGI1‐Ab‐E, especially in the emerging era of clinical trials within AE.^13^, ^14^ Here, we aimed to develop a LGI1‐Ab‐E‐specific PROM to distil the core patient‐reported symptoms, and how each impacts QoL and activities of daily living (ADL). We anticipated that capturing ‘symptom burden’ longitudinally from around 3 to 6 months post‐treatment initiation would provide an especially useful tool for future clinical studies.

## Methods

### Participant identification

Participants were recruited via the Oxford Autoimmune Neurology Group (OANG) database with signed informed consent (REC16/YH/0013). Sample sizes in stages one and two (*n* = 18 and *n* = 15, respectively, excluding relatives and stakeholders) followed established guidelines^15^, were similar to other rare disease studies^16^ and of sufficient ‘power’ to identify potential issues with questions.^17^ The sample size in stage three (*n* = 66) is considered adequate for PROM development, and substantial for this disease.^18^ Study design adhered to STROBE guidelines for cross‐sectional studies.^19^

### Study planning

**Figure 1:**
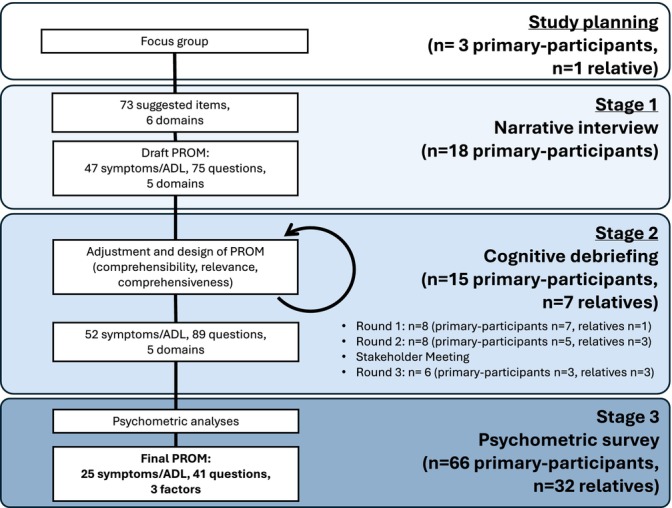
Flowchart of LANTERN design methodology and participant numbers at each stage. Participants could contribute to stages 1 or 2, but not both. But participants from both stages were invited to contribute to stage 3, in which 66/91 invitees completed the psychometric questionnaire.

The study design and feasibility were explored in a focus group, with three patients, one relative and a study group of two PROM specialists and two neurologists experts in LGI1‐Ab‐E. The project proposal (Supplemental Materials) was shared with participants: All contributors concurred with the significance and necessity of creating a LGI1‐Ab‐E patient‐need‐specific PROM, which we termed the ‘LGI1‐ANTibody Encephalitis RatiNg’ scale (LANTERN), and provided early insights on interview themes. Thereafter, the LANTERN was designed using a three‐stage mixed‐methods approach, aligned with FDA guidelines for PROM development (Fig. 1).^20^

### Stage 1. Item generation

To gather more detailed personal narratives than in focus groups, semi‐structured interviews were conducted with 18 primary participants (i.e. who had experienced LGI1‐Ab‐E). Full details of interview structure, determination of sample size and data saturation and generation of themes are contained in supplemental methods. In brief, semi‐structured interviews were conducted, recorded and transcribed, exploring all symptoms experienced since the onset of illness. Thematic analysis identified was used to generate themes and domains from which preliminary questions were drafted (File S1).

Based on insights from interviews, two distinct question types were drafted, as per guidelines.^18^, ^20^ For disease‐related symptoms, one addressed symptom frequency and the other addressed the impact of that symptom on QoL. Previously, this method has proven sensitive in longitudinal measures of symptom burden in other neurological disorders.^21^ Another subset of questions addressed ADL, and how these are affected by the illness more broadly. The remaining components of the questionnaire were carefully adjusted: text, layout, instructions, recall period and response options. The decision to use a 5‐item Likert response scale was made after reviewing current measures^12^, ^22^, ^23^, ^24^ and achieving a balance between reliability and ease‐of‐completion.^25^ Clinical opinion and review of existing measures^22^, ^23^, ^26^, ^27^, ^28^ also informed the decision to use a 4‐week‐recall period, perceived to provide an accurate window of the current status while reducing variability associated with shorter recall periods. Response options were later tested in the cognitive debriefing interviews (Stage 2).

### Stage 2. Initial item selection

**Figure 2:**
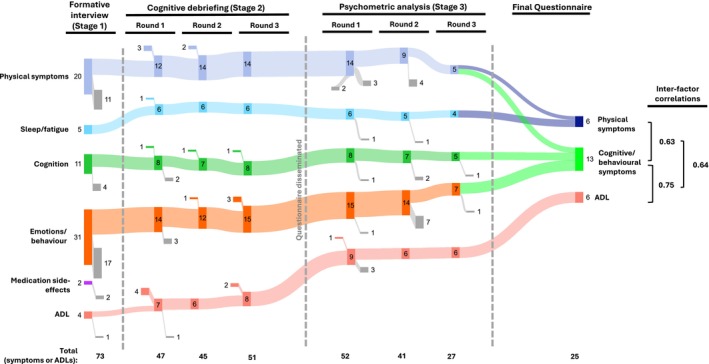
Item development process. Number of symptoms and ADL items at each stage of the LANTERN development process, grouped by theoretical domains. These are ultimately distilled to three final factors with inter‐factor Spearman correlation coefficients shown. The number of new symptoms/ADL added to each domain at each stage is shown, as are those removed (in grey). Two symptoms (weight loss and increased interest in sex) symptoms were removed prior to psychometric analysis (stage 3) due to inconsistencies with their ‘inverse symptoms’ (i.e. weight gain and reduced interest in sex, *n* = 11 and *n* = 12 inconsistencies, respectively). While it is possible that both could be present over the course of an illness, this is unlikely within a 4‐week recall period. The removed symptoms were less frequent and less impactful than their counterparts and felt to be less clinically relevant. In the first round of psychometric analysis, 5/41 symptoms and three of nine ADL were removed due to floor effect, overlap with other items and/or poor loading on factor analysis. In round two, 14/36 symptoms were removed due to high correlation and/or poor loading. In round three, one symptom (obsessiveness) was removed due to poor factor loading and another (arranging past events in a timeline) because of high correlations (>0.65, Spearman) with long‐term memory, which were not felt to be sufficiently distinct. This left a two‐factor symptom‐burden subscale of 19 symptoms (35 questions) and a single‐factor ADL subscale of 6 questions.

To evaluate preliminary LANTERN content validity and to eliminate redundant items, draft questionnaires were presented individually to a second cohort. Each question was tested on a total of 15 primary participants over three rounds (including more than five participants in the first round, and at least three in subsequent rounds), as per guidelines,^15^, ^18^ and separately at a stakeholder meeting including all co‐authors. Adjustments were made to the survey after each round of cognitive debriefing (Fig. 2, File S2). Details of the chronology and criteria used to select items at this stage are included in supplemental methods.

### Stage 3. Psychometric survey

Next, additional and existing participants were invited to participate in the psychometric survey, Stage 3, to inform item reduction and psychometric properties of LANTERN. To increase inclusivity, the survey could be completed via direct (participant alone) or aided (with assistance) self‐report. Additionally, participants completed several background and clinical questions (Questionnaire S1), verified by clinical notes and other research databases. The full psychometric survey (Questionnaire S2) was provided to participants either online (via REDCap^29^, ^30^) or on paper. For each participant completing the survey, an invitation was sent for their relative to complete a similar questionnaire. These questions were identical in wording but changed to the third‐person (Questionnaire S3). Finally, 4–8 weeks later, 40 participants were invited (in order of response) to repeat the questionnaire to assess test–retest reliability alongside a visual analogue scale (VAS), to subjectively measure their post‐illness quality‐of‐life.

### Standardised validation questionnaires

Participants were asked to complete standardised questionnaires shown in previous LGI1‐Ab‐E studies to capture deficits and cover the main themes which arose from Stages 1–2. These included the mRS,^8^ EQ‐5D‐5L health‐related QoL,^24^ Hospital anxiety and Depression score (HADS),^12^ Modified Fatigue Impact Scale (MFIS),^11^ Neuro‐QOL‐Emotional and Behavioural Dyscontrol,^31^ and Neuro‐QOL SF v2.0‐Cognitive Function.^26^ We hypothesised each measure would correlate with at least one of the final factors (Spearman rho >0.5/<−0.5).

### Psychometric analyses

For each symptom, scores from corresponding frequency and severity questions were multiplied to create a single product score, representing the overall symptom burden. In some cases, the same ‘impact’ question was used for more than one symptom (e.g. overall impact of fatigue on QoL divided by how often physical and mental fatigue are experienced). In such cases, frequency scores were multiplied by the same impact score to create two product scores. Psychometric analyses of the ADL subscale were performed separately.

Spearman correlations were performed to identify the highest correlations between symptoms/ADL. Given the modest sample size, confirmatory factor analyses were not performed. Instead, exploratory factor analyses with oblimin rotation assessed the structural validity of LANTERN, based on 1‐ to 5‐factor solutions, as suggested by factor diagnostics including parallel analysis, very simple structure, Velicer minimum average partial test and Bayesian Information Criterion. While there were no absolute criteria for removal, this was considered if symptoms/ADL met any of the following criteria:

- Floor or ceiling effect (>95% of respondents scoring <2 or ≥2).
- High Spearman correlation with another symptom/ADL (0.8 in round one, 0.7 in round two, 0.65 in round three).
- Poor loading (<0.5) in factor analysis (round two onwards).

Using these criteria, repeat psychometric analyses were performed until no more were removed.

Cronbach‐alpha scores were calculated for the final factors, as a measure of internal consistency reliability. Final factors scores were normalised to a 0–100 scale. A normalised single sum score of all these factors with equal weighting was computed. Intraclass correlation coefficients (ICC) for test–retest reliability used a one‐way random effect model,^32^ and Spearman correlation coefficients assessed other correlations. Missing data were excluded. Analyses were performed in R version 4.3 using packages *psych*, *psychTools* and *lavaan*. Figures were produced using R and sankeymatic.com.

## Results

Figures 1, 2 and Table 1 show the participant numbers, demographics and clinical characteristics at each stage of the LANTERN development process.

**Table 1:** Demographics and assessments of participants at the three stages of LANTERN development.

	Stage 1 (*N* = 18)	Stage 2 (*N* = 15)	Stage 3 (*N* = 66)	*p*‐value
	Median (range), unless otherwise stated			
Sex (*N*, female, %)	5/18 (27.8%)	4/15 (26.7%)	15/66 (22.7%)	0.83
Age (Years)	67 (40–86)	70 (53–85)	68 (41–87)	0.46
Age at onset (Years)	61 (39–83), *N* = 18	68 (45–83), *N* = 15	63 (39–83), *N* = 65	0.51
Months since onset	36.5 (5–152), *N* = 18	39 (12–132), *N* = 15	57.5 (9.5–195.9), *N* = 65	0.12
Months since diagnosis	30 (1–128), *N* = 17	30 (9–132), *N* = 14	51.5 (8.5–181.5), *N* = 65	0.05
On IT (%)	11/18 (61.1%)	7/15 (46.7%)	16/65 (24.6%)	0.009**
Months since first IT	27 (2–133), *N* = 18	32 (9–132), *N* = 15	51.5 (8.5–181.5), *N* = 63	0.14
Months since last IT (if completed)	21 (1–47), *N* = 6	50.5 (1–121), *N* = 8	21.7 (−15.5–132.5), *N* = 46	0.34
Ever relapsed (%)	4/18 (22.2%)	2/14 (14.3%)	15/44 (34.1%)	0.34
mRS	1 (0–4), *N* = 18	2 (1–4), *N* = 11	2 (0–3), *N* = 61	0.048
ACE‐III	94 (85–99), *N* = 18	92 (76–99), *N* = 11	N/A	0.3
FAB	16.5 (14–18), *N* = 18	16 (12–18), *N* = 8	N/A	0.38
CASE	1.5 (0–3), *N* = 16	2 (0–8), *N* = 10	2 (0–5), *N* = 61	0.17
EQ5D5L‐VAS	77.5 (10–100), *N* = 16	60 (33–94), *N* = 9	75 (11–100), *N* = 64	0.25
HADS‐A	6 (1–11), *N* = 15	3.5 (0–12), *N* = 10	4 (0–16), *N* = 65	0.5
HADS‐D	3 (0–9), *N* = 15	5 (1–12), *N* = 10	5 (0–14), *N* = 65	0.36
MFIS	19.5 (0–66), *N* = 14	44 (4–73), *N* = 10	30 (0–84), *N* = 66	0.25

Post hoc analyses with Bonferroni correction identified statistically significant** differences in proportion on IT between stages 1 and 3 (*p* =0.025, chi‐square test) and in mRS between stages 1 and 2 (*p* = 0.08, Dunn test). ACE‐III and FAB could not be performed for stage three as measures in this stage were only performed by questionnaire over the phone. *p*‐values computed by Fisher's exact test (Sex, ever relapsed), Chi‐square test (On IT), Mann–Whitney U (ACE‐III, FAB), and Kruskal‐Wallis (others).

ACE‐III, Addenbrooke's Cognitive Examination‐III; CASE, Clinical Assessment Scale for Encephalopathy; EQ5D5L‐VAS, EuroQol‐5 Dimensions‐5 Levels Visual Analogue Scale; FAB, Frontal assessment battery; HADS, Hospital Anxiety and Depression Scale; IT, Immunotherapy; MFIS, Modified Fatigue Impact Scale; mRS, Modified Rankin Scale.

### Stage 1. Item generation

To highlight participant quotes (Table S1) and identify key symptoms, 18 participants took part in semi‐structured narrative interviews, of a median duration of 41 min. From 73 items, the thematic analysis identified six domains (Fig. 2). Four domains related to symptoms of LGI1‐Ab‐E: Physical, Sleep/fatigue, Cognition and Emotions/behaviour, consistent with our previous patient surveys.^6^, ^7^ Two sets of questions were generated to address both the frequency of these symptoms and their intensity (impact on QoL).^21^ A fifth domain—ADL—captured how the illness more broadly affects the day‐to‐day life of participants: a separate subsection with one question per ADL was developed for this domain. One domain (‘medication side‐effects’) was removed, as these items either overlapped with other symptoms or were not easily distinguished from disease‐related symptoms. In total, 47 symptoms/items (40 symptoms and seven ADL; 77 questions) were included in the first draft. This was reviewed with the study team and consensus was achieved before proceeding. All items which arose from interviews, and the rationale for removing or altering them, are presented (File S1).

### Stage 2. Initial item selection

Fifteen primary‐participants (who had not participated in Stage 1) and seven relatives contributed to three rounds of cognitive debriefing aimed at fine‐tuning the psychometric survey (Fig. 1). After each round, changes were made to content and wording of questions, instructions to participants and questionnaire layout (Fig. 2; File S2).

Most participants approved the agreed wording of the instructions (82%), the response options using the 5‐point Likert scale (77%) and the 4‐week‐recall period (77%), as well as the ordering of items (100%). However, multiple adjustments to the question wording were made (74% after Round 1, 34% after Round 2, 18% after Round 3 and stakeholder meeting; File S2). Ten new symptoms/ADL were added based on participant suggestions and six were removed (Fig. 2; File S2). Thereafter, participants confirmed the questionnaire to be comprehensive and relevant.

After cognitive debriefing, and with the consensus of the study group reached in a stakeholder meeting, a psychometric survey with 43 symptoms (80 questions) and nine ADL (nine questions) was taken forward to Stage 3 (Fig. 2; File S3).

### Stage 3. Psychometric survey and final item selection

The psychometric survey was completed by 66 participants (*n* = 63 online, *n* = 3 by post; 91 were invited and the remainder did not respond). 22/66 (33%) were completed by assisted direct report, and 32/66 relatives completed a proxy version of the questionnaire (Questionnaire S3). The survey took participants an average of 14 minutes to complete (range 1–33 where captured, *n* = 45/66). 19/40 participants completed the questionnaire again after a median of 5.6 weeks (range 4.9–9.0). Next, the data were checked for errors: two symptoms (weight loss and increased interest in sex) were removed prior to detailed psychometric analysis, as frequent inconsistencies were identified where both these and their inverse symptoms were reported by participants (i.e., weight gain and reduced interest in sex, *n* = 11 and *n* = 12 inconsistencies, respectively).

Three rounds of psychometric analyses were performed on the remaining questions. After the removal of confounding or co‐dependent symptoms at each stage, the EFA was re‐estimated to assess the effects on remaining symptoms/ADL. In total, the number of symptoms/ADL was halved from 50 to 25 over three rounds, leaving the final two‐factor symptom‐burden score of 19 symptoms (6 physical symptoms—10 questions and 13 cognitive/behavioural symptoms—25 questions) and a single‐factor ADL score of six questions (Fig. 2, File S3).

**Figure 3:**
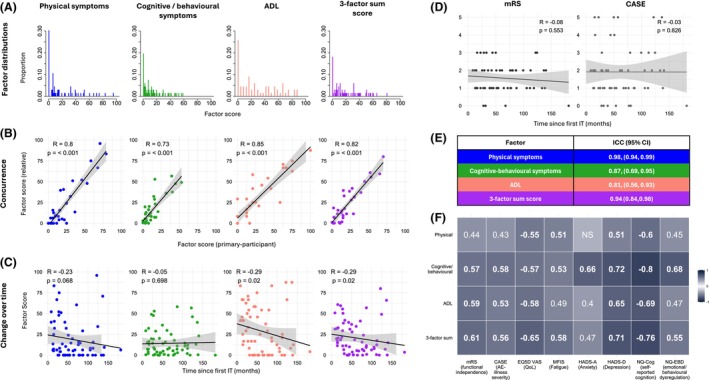
LANTERN sensitivity analyses. (A) Histograms of participant scores for all three factors, normalised to a 0–100 scale and for a three‐factor sum score, where the three‐factor scores are equally weighted and again normalised. (B) Factor scores from questionnaires completed by relatives correlated very highly with those completed by participants, providing support for both the applicability of these factors and the reliability of primary participants. (C) ADL show a modest but statistically significant inverse correlation with time since the first immunotherapy (IT) in this cross‐sectional cohort, where most participants (50/66) were no longer on IT and the same association was not with current standard measures (mRS and Case; D). (E) Test–retest analysis demonstrates good‐to‐excellent intraclass correlation coefficients (ICC) for all factors and the three‐factor sum score. (F) As hypothesised each factor correlates with at least one of a series of other validated measures, specifically chosen to capture specific domains important to participants. CASE, Clinical Assessment of Severity in Autoimmune Encephalitis; EQ5D5L‐VAS, EuroQol‐5 Dimensions‐5 Levels Visual Analogue Scale; HADS, Hospital Anxiety and Depression Scale; MFIS, Modified Fatigue Impact Scale; mRS, Modified Rankin Scale; NQ‐Cog, Neuro‐QOL SF v2.0‐Cognitive Function; NQ‐EBD, Neuro‐QOL‐Emotional and Behavioural Dyscontrol; or more general measures of health and quality‐of‐life. Heatmap displays Spearman correlations. Those greater than 0.5 or less than −0.5 are highlighted in bold, and one adjusted *p*‐value >0.05 is displayed as not significant (NS).

Each of the final factors demonstrated good internal reliability with Cronbach‐alpha scores: 0.90 for physical symptoms; 0.91 for cognitive/behavioural symptoms; and 0.85 for ADL (Table 2). As there was a strong correlation between all three factors (>0.6, Fig. 2), a final sum score was calculated weighting each of the factors equally, from 0 to 100, herein referred to as the ‘sum score’ (Fig. 3A). This overall measure of disease burden also demonstrated strong internal reliability (Cronbach‐alpha 0.86; Table 2).

**Table 2:** Items and factor analysis.

Factor	Description	Loadings on EFA		Cronbach alpha (for factor)
		Factor 1	Factor 2	
Physical symptoms	Mental fatigue	0.80		0.90
	Physical fatigue	0.94		
	Weakness mobilising outside the house	0.79		
	Requiring assistance with day‐to‐day activities	0.62		
	Problems sleeping	0.69		
	Sleeping too much	0.65		
Cognitive/behavioural	Focal seizures		0.45	0.91
	Generalised seizures		0.46	
	Problems concentrating		0.77	
	Weight gain		0.45	
	Short‐term memory problems		0.74	
	Problems with directions		0.79	
	Problems making decisions		0.69	
	Feeling overly emotional		0.72	
	Long‐term memory problems		0.66	
	Low mood		0.70	
	Anxiety		0.76	
	Short temper		0.58	
	Interest in things previously enjoyed		0.52	
ADL	Job	0.28	NA	0.85
	Activities previously enjoyed	0.76	NA	
	Driving	0.76	NA	
	Getting around outside the house	0.90	NA	
	Household chores	0.83	NA	
	Being a burden on others	0.71	NA	
Three‐factor sum score				0.86

Table of item descriptions, Exploratory factor analysis (EFA) factor loadings, Cronbach alphas, and final factors. Factor loadings are displayed for the two‐factor EFA of symptoms (dark grey) and single‐factor EFA of activities of daily living (ADL). Loadings below 0.3 are suppressed.

### Concurrence with relative questionnaires

Thirty‐two participants had near‐identical questionnaires completed by a relative (spouses/partners, family members, or close friend) (Files S3a and S3b), and each relative‐completed factor score showed strong correlations with the primary‐participant score: Physical symptoms; *R* = 0.8, cognitive/behavioural symptoms; 0.73, ADL; 0.85 (*p* < 0.001 in all cases, Fig. 3B).

### Time from disease onset

To inform the potential value of LANTERN at different time points from diagnosis, we hypothesised factor scores would reduce from immunotherapy administration, and reflect cross‐sectional clinical improvements. One of the three factors (ADL), plus the sum score, showed statistically significant decreases with time from immunotherapies, while physical‐symptom burden also trended towards this relationship (physical symptoms; *R* = −0.23, *p* = 0.068, cognitive/behavioural symptoms; *R* = −0.05, *p* = 0.698, ADL and Sum score; *R* = −0.29, *p* = 0.02; Fig. 3C). Hence, some factors demonstrate sensitivity to stage of illness in this cross‐sectional cohort. Notably, mRS and CASE scores demonstrated no correlation with time since the first immunotherapy (*R* = −0.08, *p* = 0.553 and *R* = −0.03, *p* = 0.826, respectively. Fig. 3D), suggesting this cross‐sectional cohort may represent a range of outcome severities that have plateaued, rather than one experiencing ongoing improvement.

### Test–retest and standardised questionnaire comparisons

Of the 19/40 invited participants who repeated the questionnaire, two were excluded as they demonstrated a change in VAS score of >1 SD from the mean (test–retest reliability requires clinical stability). The ICC for all three factors demonstrated good‐to‐excellent test–retest reliability: Physical symptoms; 0.98, (95%CI 0.94, 0.99); cognitive/behavioural symptoms; 0.87, (95%CI 0.69, 0.95), ADL; 0.81 (95%CI 0.56–0.93) (Fig. 3E).

**Figure 4:**
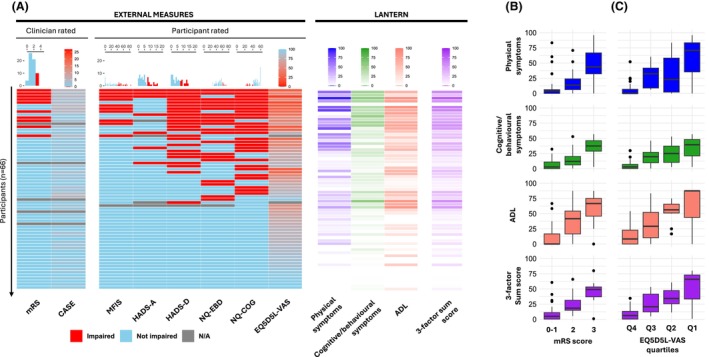
Individualised patient data from LANTERN and external measures. (A) Heatmap with each row denoting one study participant, with their results across both external measures (left; both clinician and patient‐rated, sorted in descending order of number of impaired domains) and the LANTERN (right). The CASE and EQ5D5L‐VAS are shown on continuous scales, whereas external measures with clinically defined cut‐offs for impairment are illustrated as binary variables, with histograms above illustrating the distributions of overall scores (also Table S2). Each of the LANTERN factors and the three‐factor sum score are illustrated as continuous variables. Missing data are highlighted in grey. The discriminatory ability of the LANTERN is further seen by comparing median scores against current measures of function (mRS, B) and quality‐of‐life (EQ5D5L‐VAS, descending quartiles (C). CASE, Clinical Assessment of Severity in Autoimmune Encephalitis; EQ5D5L‐VAS, EuroQol‐5 Dimensions‐5 Levels Visual Analogue Scale; HADS, Hospital Anxiety and Depression Scale; MFIS, Modified Fatigue Impact Scale; mRS, Modified Rankin Scale; NQ‐Cog, Neuro‐QOL SF v2.0‐Cognitive Function; NQ‐EBD, Neuro‐QOL‐Emotional and Behavioural Dyscontrol; Q1‐Q4, Quartiles of 0–100 continuous scale.

Next, each factor score and the three‐factor sum score were compared against a selection of health‐related clinician‐ and patient‐reported questionnaires which both reflect known features of LGI1‐Ab‐E and symptoms/ADL broadly identified throughout Stages 1 and 2 (Figs. 3F and 4; Table S2). 31/32 direct comparisons showed statistically significant correlations, and in 24/32 the Spearman correlation coefficient was >0.5, suggesting concordant validity. Reassuringly, questionnaires assessing cognition (Neuro‐QOL SF v2.0‐Cognitive Function) and low mood (HADS‐D) demonstrated the highest correlations with the cognitive/behavioural symptom factor, suggesting the LANTERN has strong internal validity and captures disease‐relevant features. However, imperfect correlations suggest LANTERN could provide additional value over conventional questionnaires.

Indeed, direct comparisons with LANTERN show a marked insensitivity of current measures, with only 10/67 participants considered ‘impaired’ using mRS and none scoring >5/27 on CASE. Whereas >50% demonstrated deficits in at least one other validated questionnaire (fatigue, mood, behaviour or cognition) which, alongside the EQ‐5D‐5L VAS QoL assessment, were more comparable to the LANTERN scores (Fig. 4A). Nevertheless, epochs of mRS and EQ‐5D‐5L VAS showed quantitative relationships with all four domains within LANTERN (Fig. 4B,C).

## Discussion

The development of a disease‐specific PROM for LGI1‐Ab‐E (LANTERN) marks a major step in improving several contemporary clinical and research challenges in the assessment of people with this condition. Despite frequently stated ‘good’ recoveries based on clinician assessments, LGI1‐Ab‐E has a significant impact on patient‐rated QoL.^6^, ^7^ LANTERN fills this critical gap by providing a patient‐rated tool for measuring both symptom frequency and impact on QoL. Following in‐depth psychometric analyses, LANTERN distils patient‐reported outcomes into a comprehensive 41‐question summary, with two subsections and three factors, which can typically be completed in under 10 min. It performs well over the subacute‐to‐chronic disease course in concordant validity, both between assessments and between relatives and patients and in discriminatory ability, providing increased variance compared to conventional measures. Also, LANTERN captures key features which are prevalent and troublesome to patients but under‐recognised by non‐specific clinician‐rated measures.^6^, ^7^ LANTERN is likely to have significant utility in research and patient‐care settings, by providing a more disease‐specific and nuanced clinical trial outcome measure and by identifying the key features for clinicians to focus on in clinic.

Important aspects of this measure are its adherence to FDA guidelines and the rigorous, participant‐driven mixed‐methods approach used to identify *a priori* the most impactful features of illness identified by those who have experienced LGI1‐Ab‐E. By conducting repeated in‐depth one‐to‐one interviews with participants we ensured that questionnaire design and content were comprehensive and easily interpretable, before circulation to a broader cohort. The psychometric results of this final step allowed us to reduce the number of questions by over 50% while maintaining sensitivity. Further, to guarantee the validity and reliability of the LANTERN, we took a number of important steps/stages during the development process in alignment with FDA guidelines and the existing literature.^15^, ^18^, ^20^ People with LGI1‐Ab‐E and their families were involved in the project design from inception and questionnaires completed by relatives corroborated the answers by primary‐participants, with good‐to‐excellent test–retest reliability across all factors. Significant correlations between LANTERN's factor scores and several existing, assumed measures (including the current standards; mRS and CASE), suggest LANTERN not only captures variance within these measures but also additional clinical features.

Limitations of this study include that LANTERN, and most PROMs, may not be as useful in the acute disease phase, where amnestic or delirious patients cannot reliably complete the questionnaire. Also, the small numbers limit the scope of feasible psychometric tests,^16^ although inevitable in a rare disease. Further, while we observed a moderate association between treatment duration and ADL score, this association was not seen with symptom scores, mRS or CASE. Correlations with a panel of functional measures and validated questionnaires were more robust, suggesting that this cross‐sectional study of a largely convalescent cohort is not best positioned to capture the true longitudinal responsiveness of the LANTERN. We expect the LANTERN can be reliably administered as early as 3 months into treatment, once patients are responding to therapy and are physically capable of completing the questionnaire, then onwards at 3–6 month intervals. Longitudinal assessments of this nature will likely reveal time‐dependent improvements in LANTERN score, particularly over the first 2 years of illness when the greatest functional gains in AE are typically observed.^33^

It is also possible that some symptoms are being confounded by current first‐line therapies (e.g. fatigue and corticosteroid‐induced weight gain). We included these symptoms as they can be difficult to parse from disease‐related symptoms, are problematic to people with LGI1‐Ab‐E, and may be addressed in future trials of steroid‐sparing agents.

The LANTERN was designed to have two separate subscores (symptom‐burden and ADL‐impact). EFA directed us towards a two‐factor symptom‐burden score and a single‐factor ADL score. We, therefore, recommend in practice that these factors are interpreted individually. Yet, we also present results of a single sum score of the overall disease burden, where all factors are equally weighted, which also demonstrates strong internal validity and performs well in sensitivity analyses. This single score may be more easily interpretable to the user, but there are drawbacks to combining differently designed measures and assuming equal weighting. These may be addressed by investigating the applicability and generalisability of LANTERN in another cohort, including prospective recruitment with repeated measures over time to identify the minimum clinically important difference, cross‐cultural adaptation and comparison against other neurological illnesses.

In summary, the LANTERN is a novel symptom‐burden PROM in LGI1‐Ab‐E with demonstrable initial content, structural and construct validity, and internal and test–retest reliability. LANTERN has been designed for use in clinical practice and in research, including clinical trials, to estimate symptom burden over time and at key points throughout treatment and recovery. Future validation of its utility is awaited.

## Conflict of Interest

SB is named on a patent application entitled ‘Diagnostic Strategy to improve specificity of CASPR2 antibody detection’ ‘(TBA / BB Ref. JA94536P.GBA)’. AEH has received grant support from UCB and consultancy payment from Argenx. SRI has received honoraria/research support from UCB, Immunovant, MedImmun, Roche, Janssen, Cerebral therapeutics, ADC therapeutics, Brain, CSL Behring and ONO Pharma, and receives licenced royalties on patent application WO/2010/046716 entitled ‘Neurological Autoimmune Disorders’, and has filed two other patents entitled ‘Diagnostic method and therapy’ (WO2019211633 and US app 17/051,930; PCT application WO202189788A1) and ‘Biomarkers’ (WO202189788A1, US App 18/279,624; PCT/GB2022/050614).

## Author Contributions

Conception and study design: BW, MJK, PAP, SRI. Data acquisition and analysis: BW, MJK, BC, AYL, JS. Drafting manuscript and figures: BW, MJK, BC, AYL, AH, JS, SB, CS, PAP, SRI.

## Supporting information


File S1.

File S2.

**File S3a**.

**File S3b**.

**Questionnaire S1**.

**Questionnaire S2**.

**Questionnaire S3**.

**Questionnaire S4**.

Data S1.

Table S1.

## Data Availability

Anonymised data from this study may be shared upon reasonable request to the corresponding author from a qualified investigator with ethical approval.
