# Supplementary material for: Capturing what matters: Patient‐reported LGI1‐ANTibody encephalitis outcome RatiNg scale (LANTERN)
Source: Ann Clin Transl Neurol. 2025 Feb 25;12(4):821–31. doi: 10.1002/acn3.70006 (PMC12040509; doi:10.1002/acn3.70006)
Supplement: Supplementary file 5 — Questionnaire S1. [file ACN3-12-821-s007.pdf]

Personal information

Please complete the survey below.Thank you!

1) Age

____

2) Sex

◯ Female
◯ Male
◯ Prefer not to say

3) If your gender is different to the sex you were assigned at birth, please enter details here.

____

4) Onset of encephalitis symptoms (Month/Year)

____

5) Date of encephalitis diagnosis (Month/Year)

____

6) Did you receive any of the following forms of immunotherapy (treatments that affect the immune system)?
Select all that apply.

☐ Intravenous high dose Steroids (Methylprednisolon)
☐ Oral Steroids (Prednisolon)
☐ Plasma exchange
☐ IVIG
☐ Rituximab
☐ Mycophenolate
☐ Azathioprine
☐ I don't know

7) Date of first immunotherapy, if known (Month/Year)

____

8) Date of last dose of immunotherapy, if known (Month/Year)

____

9) Did you have a relapse?

◯ Yes
◯ No
◯ I don´t know

10) When was your relapse? (Month/Year?)

____

11) Did you receive anti-seizure medication at any stage? (For example Epilim, Keppra, Levetiracetam, Lamotrigine, Lacosamide)?

◯ Yes
◯ No
◯ I don´t know

12) Are you still on anti-seizure medication?

◯ Yes
◯ No
◯ I don´t know

13) What was your employment status before your encephalitis?

◯ In part-time employment
◯ In full-time employment
◯ Retired
◯ Studying
◯ Other

14) What is your employment status NOW?
- ◯ Employed (same role)
- ◯ Employed (reduced role)
- ◯ Retired due to encephalitis
- ◯ Retired (unrelated to encephalitis)
- ◯ Studying
- ◯ Other

15) If you selected "other" in the previous question, describe here

____

16) What was your profession before your encephalitis?

____

17) Did you have to change your role in occupation (retirement or change of position)?
- ◯ Yes
- ◯ No
- ◯ Not applicable

18) If yes, how did your role change?

____

19) Did you have to change the number of hours you were working per week?
- ◯ Yes
- ◯ No
- ◯ Not applicable

20) If yes, to what degree?
- ◯ Less than 25%
- ◯ 26-50%
- ◯ 51-75%
- ◯ More than 75%
- ◯ 100% (retired due to encephalitis)

21) [survey-duration-completed]

____
