# Supplementary material for: Capturing what matters: Patient‐reported LGI1‐ANTibody encephalitis outcome RatiNg scale (LANTERN)
Source: Ann Clin Transl Neurol. 2025 Feb 25;12(4):821–31. doi: 10.1002/acn3.70006 (PMC12040509; doi:10.1002/acn3.70006)
Supplement: Supplementary file 6 — Questionnaire S2. [file ACN3-12-821-s001.pdf]

Questionnaire for LGI1 encephalitis

Please read every question below and select the answer that best applies to you in the past 4 weeks, even if it may be caused by a different co-existing disease.

PHYSICAL SYMPTOMS

These questions ask HOW OFTEN you experience physical symptoms.

IN THE PAST 4 WEEKS...

		Almost never/Never	Rarely	Sometimes	Often	Almost always/Always
1)	I had focal (small) seizures lasting only seconds to a few minutes (e.g. recurrent feelings of goosebumps, shivers, arm/leg/face twitches, etc.).	◯	◯	◯	◯	◯
2)	I had generalised/tonic-clonic (big) seizures or fits (e.g with shaking and loss of consciousness).	◯	◯	◯	◯	◯
3)	Weakness or unsteadiness limited my mobility around the house.	◯	◯	◯	◯	◯
4)	Weakness or unsteadiness limited my mobility outside the house (e.g. going to the shops).	◯	◯	◯	◯	◯
5)	Weakness or unsteadiness limited me when I tried to exercise.	◯	◯	◯	◯	◯
6)	I had problems with coordination of my hands/arms (performing controlled movements).	◯	◯	◯	◯	◯
7)	I experienced painful tingling, burning or pins and needles.	◯	◯	◯	◯	◯
8)	I experienced joint pain.	◯	◯	◯	◯	◯
9)	I needed assistance in day-to-day activities.	◯	◯	◯	◯	◯
10)	I needed to have someone looking after me or with me.	◯	◯	◯	◯	◯
11)	I noticed I had gained weight.	◯	◯	◯	◯	◯
12)	I noticed I had lost weight	◯	◯	◯	◯	◯
13)	My interest in sex was reduced.	◯	◯	◯	◯	◯
14)						

My interest in sex was increased.	○	○	○	○	○

PHYSICAL SYMPTOMS

These questions ask how your physical symptoms affect your QUALITY-OF-LIFE.

IN THE PAST 4 WEEKS....

		Not at all / symptom not present	Mildly	Moderately	Severely	Extremely
15)	Seizures impacted the quality of my life.	○	○	○	○	○
16)	Weakness or unsteadiness impacted the quality of my life.	○	○	○	○	○
17)	Coordination problems of hands/arms impacted the quality of my life.	○	○	○	○	○
18)	Painful tingling, burning, or pins and needles impacted the quality of my life.	○	○	○	○	○
19)	Joint pain impacted the quality of my life.	○	○	○	○	○
20)	Needing assistance in day-to-day activities impacted the quality of my life.	○	○	○	○	○
21)	Needing someone to look after me or with me impacted the quality of my life.	○	○	○	○	○
22)	Weight gain impacted the quality of my life.	○	○	○	○	○
23)	Weight loss impacted the quality of my life	○	○	○	○	○
24)	Having a reduced interest in sex impacted the quality of my life.	○	○	○	○	○
25)	Having an increased interest in sex impacted the quality of my life.	○	○	○	○	○

SLEEP AND FATIGUE

These questions ask HOW OFTEN you experience sleep and fatigue symptoms.

IN THE PAST 4 WEEKS...

		Almost never/Never	Rarely	Sometimes	Often	Almost always/Always
26)	I had problems sleeping at night.	◯	◯	◯	◯	◯
27)	I slept too much.	◯	◯	◯	◯	◯
28)	I was still tired after a night's sleep.	◯	◯	◯	◯	◯
29)	I had vivid or bad dreams.	◯	◯	◯	◯	◯
30)	I felt tired or fatigued after physical activity (e.g. doing daily chores, walking, exercise).	◯	◯	◯	◯	◯
31)	I felt tired or fatigued after mental activity (e.g. when trying to concentrate for a while).	◯	◯	◯	◯	◯

SLEEP AND FATIGUE

These questions ask how your sleep and fatigue symptoms affect your QUALITY-OF-LIFE.

IN THE PAST 4 WEEKS....

	Not at all / symptom not present	Mildly	Moderately	Severely	Extremely
32) Sleep problems impacted the quality of my life.	○	○	○	○	○
33) Vivid or bad dreams impacted the quality of my life.	○	○	○	○	○
34) Feelings of tiredness or fatigue impacted the quality of my life.	○	○	○	○	○

MEMORY AND COGNITION

These questions ask HOW OFTEN you experience memory and cognition symptoms.

IN THE PAST 4 WEEKS...

		Almost never/Never	Rarely	Sometimes	Often	Almost always/Always
35)	I had difficulty with short-term memory, remembering recent events (e.g. appointments, topics of conversation, etc.).	◯	◯	◯	◯	◯
36)	I had difficulty with long-term memory, remembering certain events in my past (e.g. weddings, funerals, holidays, etc.) that may have happened years before the illness.	◯	◯	◯	◯	◯
37)	I could remember events but could not put them into a timeline, (e.g. whether it was days or years ago).	◯	◯	◯	◯	◯
38)	I had problems with directions (e.g. finding a parked car, losing my way in a familiar place).	◯	◯	◯	◯	◯
39)	I had trouble concentrating (e.g. following a movie, book or conversation).	◯	◯	◯	◯	◯
40)	I had difficulty multi-tasking.	◯	◯	◯	◯	◯
41)	I had difficulty with complex tasks, e.g. following a recipe or instruction manual.	◯	◯	◯	◯	◯
42)	I had difficulty in writing or spelling.	◯	◯	◯	◯	◯

MEMORY AND COGNITION

These questions ask how your memory and cognition symptoms affect your QUALITY-OF-LIFE.

IN THE PAST 4 WEEKS....

		Not at all / symptom not present	Mildly	Moderately	Severely	Extremely
43)	Short-term memory problems impacted the quality of my life.	◯	◯	◯	◯	◯
44)	Long-term memory problems impacted the quality of my life.	◯	◯	◯	◯	◯
45)	Difficulties in putting events into a timeline impacted the quality of my life.	◯	◯	◯	◯	◯
46)	Problems with directions impacted the quality of my life (e.g. finding a parked car, losing my way in a familiar place).	◯	◯	◯	◯	◯
47)	Problems in concentrating impacted the quality of my life.	◯	◯	◯	◯	◯
48)	Difficulties with multi-tasking impacted the quality of my life.	◯	◯	◯	◯	◯
49)	Difficulties with complex tasks (e.g.following a recipe or instruction manual) impacted the quality of my life.	◯	◯	◯	◯	◯
50)	Difficulties in writing or spelling impacted the quality of my life.	◯	◯	◯	◯	◯

EMOTION AND BEHAVIOUR

These questions ask HOW OFTEN you experience emotional and behavioural symptoms.

IN THE PAST 4 WEEKS...

		Almost never/Never	Rarely	Sometimes	Often	Almost always/Always
51)	I felt sad or low in mood.	○	○	○	○	○
52)	I felt low in self-esteem or confidence.	○	○	○	○	○
53)	I felt embarrassed.	○	○	○	○	○
54)	I was overly emotional or cried very easily.	○	○	○	○	○
55)	I was short-tempered or impatient.	○	○	○	○	○
56)	I behaved overly friendly or direct to people.	○	○	○	○	○
57)	I felt anxious.	○	○	○	○	○
58)	I was determined or stubborn.	○	○	○	○	○
59)	I felt unmotivated. I had difficulties initiating an activity.	○	○	○	○	○
60)	I had difficulties making a decision.	○	○	○	○	○
61)	I had hallucinations (heard or saw things that were not there or did not make sense).	○	○	○	○	○
62)	I tended to obsess over things.	○	○	○	○	○
63)	I had times when I could not stop talking.	○	○	○	○	○
64)	I had less empathy and sensitivity to the needs of others.	○	○	○	○	○
65)	I had less interest in activities I used to enjoy.	○	○	○	○	○

EMOTION AND BEHAVIOUR

These questions ask how your emotional and behavioural symptoms affect your QUALITY-OF-LIFE.

IN THE PAST 4 WEEKS....

		Not at all / symptom not present	Mildly	Moderately	Severely	Extremely
66)	Feelings of sadness impacted the quality of my life.	○	○	○	○	○
67)	Feelings of low self-esteem or confidence impacted the quality of my life.	○	○	○	○	○
68)	Feelings of embarrassment impacted the quality of my life.	○	○	○	○	○
69)	Being overly emotional impacted the quality of my life.	○	○	○	○	○
70)	Being short-tempered or impatient impacted the quality of my life.	○	○	○	○	○
71)	Behaving overly friendly or direct to people impacted the quality of my life.	○	○	○	○	○
72)	Feelings of anxiety impacted the quality of my life.	○	○	○	○	○
73)	Being determined or stubborn impacted the quality of my life.	○	○	○	○	○
74)	Lack of motivation impacted the quality of my life.	○	○	○	○	○
75)	Difficulties in making decisions impacted the quality of my life.	○	○	○	○	○
76)	Hallucinations impacted the quality of my life.	○	○	○	○	○
77)	Tendency to obsess over things impacted the quality of my life.	○	○	○	○	○
78)	Being unable to stop talking impacted the quality of my life.	○	○	○	○	○
79)	Having less empathy and sensitivity to the needs of others impacted the quality of my life.	○	○	○	○	○

80)

Having less interest in activities I used to enjoy impacted the quality of my life.	○	○	○	○	○

Activities of Daily Living (ADL) in the past 4 weeks

	Not at all / not applicable	Mildly	Moderately	Severely	Extremely
81) My illness impacted my ability to do my job.	◯	◯	◯	◯	◯
82) My illness impacted my ability to do activities I used to enjoy.	◯	◯	◯	◯	◯
83) My illness impacted my ability to drive.	◯	◯	◯	◯	◯
84) My illness limited my ability to get around out of the house by myself.	◯	◯	◯	◯	◯
85) My illness limited my ability to do household chores by myself.	◯	◯	◯	◯	◯
86) My illness limited my ability to walk around the house by myself.	◯	◯	◯	◯	◯
87) My illness limited my ability to wash and dress myself.	◯	◯	◯	◯	◯
88) My illness limited my ability to attend social meetings.	◯	◯	◯	◯	◯
89) My illness made me a burden to people around me.	◯	◯	◯	◯	◯

90) How would you rate your current quality of life, taking your illness into account, on a scale from 0 to 100?

If 0 is the worst quality of life you can imagine and 100 is the best quality of life you can imagine?

0 50 100

(Place a mark on the scale above)

91) How did you fill out the above questions?

◯ On my own
◯ With someones help
