# Supplementary material for: Capturing what matters: Patient‐reported LGI1‐ANTibody encephalitis outcome RatiNg scale (LANTERN)
Source: Ann Clin Transl Neurol. 2025 Feb 25;12(4):821–31. doi: 10.1002/acn3.70006 (PMC12040509; doi:10.1002/acn3.70006)
Supplement: Supplementary file 7 — Questionnaire S3. [file ACN3-12-821-s002.pdf]

Questionnaire for Family member for LGI1 encephalitis

Please read every question below and select the answer that best applies to the person with encephalitis in the past 4 weeks, even if it may be caused by a different co-existing disease.

PHYSICAL SYMPTOMS

These questions ask HOW OFTEN they experience physical symptoms.

IN THE PAST 4 WEEKS...

		Almost never/Never	Rarely	Sometimes	Often	Almost always/Always
1)	They had focal (small) seizures lasting only seconds to a few minutes (e.g. recurrent feelings of goosebumps, shivers, arm/leg/face twitches, etc.).	○	○	○	○	○
2)	They had generalised/tonic-clonic (big) seizures or fits (e.g with shaking and loss of consciousness).	○	○	○	○	○
3)	Weakness or unsteadiness limited their mobility around the house.	○	○	○	○	○
4)	Weakness or unsteadiness limited their mobility outside the house (e.g. going to the shops).	○	○	○	○	○
5)	Weakness or unsteadiness limited them when they tried to exercise.	○	○	○	○	○
6)	They had problems with coordination of hands/arms (performing controlled movements).	○	○	○	○	○
7)	They experienced painful tingling, burning or pins and needles.	○	○	○	○	○
8)	They experienced joint pain.	○	○	○	○	○
9)	They needed assistance in day-to-day activities.	○	○	○	○	○
10)	They needed to have someone looking after them or with them.	○	○	○	○	○
11)	They noticed they had gained weight.	○	○	○	○	○
12)						

	They noticed they had lost weight	◯	◯	◯	◯	◯
13)	Their interest in sex was reduced.	◯	◯	◯	◯	◯
14)	Their interest in sex was increased.	◯	◯	◯	◯	◯

PHYSICAL SYMPTOMS

These questions ask how their physical symptoms affect your QUALITY-OF-LIFE.

IN THE PAST 4 WEEKS....

		Not at all / symptom not present	Mildly	Moderately	Severely	Extremely
15)	Seizures impacted the quality of their life.	○	○	○	○	○
16)	Weakness or unsteadiness impacted the quality of their life.	○	○	○	○	○
17)	Coordination problems of hands/arms impacted the quality of their life.	○	○	○	○	○
18)	Painful tingling, burning or pins and needles impacted the quality of their life.	○	○	○	○	○
19)	Joint pain impacted the quality of their life.	○	○	○	○	○
20)	Needing assistance in day-to-day activities impacted the quality of their life.	○	○	○	○	○
21)	Needing someone to look after them impacted the quality of their life.	○	○	○	○	○
22)	Weight gain impacted the quality of their life.	○	○	○	○	○
23)	Weight loss impacted the quality of their life	○	○	○	○	○
24)	Having a reduced interest in sex impacted the quality of their life.	○	○	○	○	○
25)	Having an increased interest in sex impacted the quality of their life.	○	○	○	○	○

SLEEP AND FATIGUE

These questions ask HOW OFTEN they experience sleep and fatigue symptoms.

IN THE PAST 4 WEEKS...

		Almost never/Never	Rarely	Sometimes	Often	Almost always/Always
26)	They had problems sleeping at night.	○	○	○	○	○
27)	They slept too much.	○	○	○	○	○
28)	They were still tired after a night's sleep.	○	○	○	○	○
29)	They had vivid or bad dreams.	○	○	○	○	○
30)	They felt tired or fatigued after physical activity (e.g. doing daily chores, walking, exercise).	○	○	○	○	○
31)	They felt tired or fatigued after mental activity (e.g. when trying to concentrate for a while).	○	○	○	○	○

SLEEP AND FATIGUE

These questions ask how their sleep and fatigue symptoms affect their QUALITY-OF-LIFE.

IN THE PAST 4 WEEKS....

		Not at all / symptom not present	Mildly	Moderately	Severely	Extremely
32)	Sleep problems impacted the quality of their life.	○	○	○	○	○
33)	Vivid or bad dreams impacted the quality of their life.	○	○	○	○	○
34)	Feelings of tiredness or fatigue impacted the quality of their life.	○	○	○	○	○

MEMORY AND COGNITION

These questions ask HOW OFTEN they experience memory and cognition symptoms.

IN THE PAST 4 WEEKS...

		Almost never/Never	Rarely	Sometimes	Often	Almost always/Always
35)	They had difficulty with short-term memory, remembering recent events (e.g. appointments, topics of conversation, etc.).	○	○	○	○	○
36)	They had difficulty with long-term memory, remembering certain events in their past (e.g. weddings, funerals, holidays, etc.) that may have happened years before the illness.	○	○	○	○	○
37)	They could remember events but not put them into a timeline, (e.g. whether it was days or years ago).	○	○	○	○	○
38)	They had problems with directions (e.g. finding a parked car, losing their way in a familiar place).	○	○	○	○	○
39)	They had trouble concentrating (e.g. following a movie, book or conversation).	○	○	○	○	○
40)	They had difficulty multi-tasking.	○	○	○	○	○
41)	They had difficulty with complex tasks, e.g. following a recipe or instruction manual.	○	○	○	○	○
42)	They had difficulty in writing or spelling.	○	○	○	○	○

MEMORY AND COGNITION

These questions ask how their memory and cognition symptoms affect their QUALITY-OF-LIFE.

IN THE PAST 4 WEEKS....

	Not at all / symptom not present	Mildly	Moderately	Severely	Extremely
43) Short-term memory problems impacted the quality of their life.	○	○	○	○	○
44) Long-term memory problems impacted the quality of their life.	○	○	○	○	○
45) Difficulties in putting events into a timeline impacted the quality of their life.	○	○	○	○	○
46) Problems with directions impacted the quality of their life (e.g. finding a parked car, losing their way in a familiar place).	○	○	○	○	○
47) Problems in concentrating impacted the quality of their life.	○	○	○	○	○
48) Difficulties with multi-tasking impacted the quality of their life.	○	○	○	○	○
49) Difficulties with complex tasks (e.g.following a recipe or instruction manual) impacted the quality of their life.	○	○	○	○	○
50) Difficulties in writing or spelling impacted the quality of their life.	○	○	○	○	○

EMOTION AND BEHAVIOUR

These questions ask HOW OFTEN they experience emotional and behavioural symptoms.

IN THE PAST 4 WEEKS...

	Almost never/Never	Rarely	Sometimes	Often	Almost always/Always
51) They felt sad or low in mood.	◯	◯	◯	◯	◯
52) They felt low in self-esteem or confidence.	◯	◯	◯	◯	◯
53) They felt embarrassed.	◯	◯	◯	◯	◯
54) They were overly emotional, cried very easily.	◯	◯	◯	◯	◯
55) They were short-tempered, impatient.	◯	◯	◯	◯	◯
56) They behaved overly friendly or direct to people.	◯	◯	◯	◯	◯
57) They felt anxious.	◯	◯	◯	◯	◯
58) They were determined or stubborn.	◯	◯	◯	◯	◯
59) They felt unmotivated. They had difficulties initiating an activity.	◯	◯	◯	◯	◯
60) They had difficulties making a decision.	◯	◯	◯	◯	◯
61) They had hallucinations (heard or saw things that were not there or did not make sense).	◯	◯	◯	◯	◯
62) They tended to obsess over things.	◯	◯	◯	◯	◯
63) They had times when they could not stop talking.	◯	◯	◯	◯	◯
64) They had less empathy and sensitivity to the needs of others.	◯	◯	◯	◯	◯
65) They had less interest in activities they used to enjoy.	◯	◯	◯	◯	◯

EMOTION AND BEHAVIOUR

These questions ask how their emotional and behavioural symptoms affect their QUALITY-OF-LIFE.

IN THE PAST 4 WEEKS....

		Not at all / symptom not present	Mildly	Moderately	Severely	Extremely
66)	Feelings of sadness impacted the quality of their life.	○	○	○	○	○
67)	Feelings of low self-esteem or confidence impacted the quality of their life.	○	○	○	○	○
68)	Feelings of embarrassment impacted the quality of their life.	○	○	○	○	○
69)	Being overly emotional impacted the quality of their life.	○	○	○	○	○
70)	Being short-tempered or impatient impacted the quality of their life.	○	○	○	○	○
71)	Behaving overly friendly or direct to people impacted the quality of their life.	○	○	○	○	○
72)	Feelings of anxiety impacted the quality of their life.	○	○	○	○	○
73)	Being determined or stubborn impacted the quality of their life.	○	○	○	○	○
74)	Lack of motivation impacted the quality of their life.	○	○	○	○	○
75)	Difficulties in making decisions impacted the quality of their life.	○	○	○	○	○
76)	Hallucinations impacted the quality of their life.	○	○	○	○	○
77)	Tendency to obsess over things impacted the quality of their life.	○	○	○	○	○
78)	Being unable to stop talking impacted the quality of their life.	○	○	○	○	○
79)	Having less empathy and sensitivity to the needs of others impacted the quality of their life.	○	○	○	○	○

80)

Having less interest in activities they used to enjoy impacted the quality of their life.	○	○	○	○	○

Activities of Daily Living (ADL) in the past 4 weeks

		Not at all / not applicable	Mildly	Moderately	Severely	Extremely
81)	Their illness impacted their ability to do their job.	○	○	○	○	○
82)	Their illness impacted their ability to do activities they used to enjoy.	○	○	○	○	○
83)	Their illness impacted their ability to drive.	○	○	○	○	○
84)	Their illness limited their ability to get around out of the house by themself.	○	○	○	○	○
85)	Their illness limited their ability to do household chores by themself.	○	○	○	○	○
86)	Their illness limited their ability to walk around the house by themself.	○	○	○	○	○
87)	Their illness limited their ability to wash and dress themself.	○	○	○	○	○
88)	Their illness limited their ability to attend social meetings.	○	○	○	○	○
89)	Their illness made them a burden to people around them.	○	○	○	○	○

90) How would you rate their current quality of life, taking their illness into account, on a scale from 0 to 100?

If 0 is the worst quality of life you can imagine and 100 is the best quality of life you can imagine?

0 50 100

(Place a mark on the scale above)
