# Supplementary material for: Capturing what matters: Patient‐reported LGI1‐ANTibody encephalitis outcome RatiNg scale (LANTERN)
Source: Ann Clin Transl Neurol. 2025 Feb 25;12(4):821–31. doi: 10.1002/acn3.70006 (PMC12040509; doi:10.1002/acn3.70006)
Supplement: Supplementary file 8 — Questionnaire S4. [file ACN3-12-821-s005.pdf]

Questionnaire for LGI1 encephalitis

Please read every question below and select the answer that best applies to you in the past 4 weeks, even if it may be caused by a different co-existing disease.

PHYSICAL SYMPTOMS

These questions ask HOW OFTEN you experience physical symptoms.

IN THE PAST 4 WEEKS...

		Almost never/Never	Rarely	Sometimes	Often	Almost always/Always
1)	Weakness or unsteadiness limited my mobility outside the house (e.g. going to the shops).	○	○	○	○	○
2)	I needed assistance in day-to-day activities.	○	○	○	○	○
3)	I had problems sleeping at night	○	○	○	○	○
4)	I slept too much	○	○	○	○	○
5)	I felt tired or fatigued after physical activity (e.g. doing daily chores, walking exercise).	○	○	○	○	○
6)	I felt tired or fatigued after mental activity (e.g. when trying to concentrate for a while).	○	○	○	○	○

PHYSICAL SYMPTOMS

These questions ask how your physical symptoms affect your QUALITY-OF-LIFE.

IN THE PAST 4 WEEKS....

		Not at all / symptom not present	Mildly	Moderately	Severely	Extremely
7)	Weakness or unsteadiness impacted the quality of my life.	◯	◯	◯	◯	◯
8)	Needing assistance in day-to-day activities impacted the quality of my life.	◯	◯	◯	◯	◯
9)	Sleep problems impacted the quality of my life.	◯	◯	◯	◯	◯
10)	Feelings of tiredness or fatigue impacted the quality of my life.	◯	◯	◯	◯	◯

Cognitive/Behavioural Symptoms

These questions ask HOW OFTEN you experience cognitive and behavioural symptoms.

IN THE PAST 4 WEEKS...

		Almost never/Never	Rarely	Sometimes	Often	Almost always/Always
11)	I had focal (small) seizures lasting only seconds to a few minutes (e.g. recurrent feelings of goosebumps, shivers, arm/leg/face twitches, etc.).	○	○	○	○	○
12)	I had generalised/tonic-clonic (big) seizures or fits (e.g with shaking and loss of consciousness).	○	○	○	○	○
13)	I had difficulty with short-term memory, remembering recent events (e.g. appointments, topics of conversation, etc.).	○	○	○	○	○
14)	I had difficulty with long-term memory, remembering certian events in my past (e.g. weddings, funerals, holidays, etc.) that may have happened years before the illness.	○	○	○	○	○
15)	I had trouble concentrating (e.g. following a movie, book or conversation).	○	○	○	○	○
16)	I had difficulties making a decision.	○	○	○	○	○
17)	I had problems with directions (e.g. finding a parked car, losing my way in a familiar place).	○	○	○	○	○
18)	I had less interest in activities I used to enjoy.	○	○	○	○	○
19)	I felt sad or low in mood.	○	○	○	○	○
20)	I felt anxious.	○	○	○	○	○
21)	I was overly emotional or cried very easily.	○	○	○	○	○
22)	I was short-tempered or impatient.	○	○	○	○	○
23)	I noticed I had gained weight.	○	○	○	○	○

Cognitive/Behavioural Symptoms

These questions ask how your cognitive and behavioural symptoms affect your QUALITY-OF-LIFE.

IN THE PAST 4 WEEKS....

	Not at all / symptom not present	Mildly	Moderately	Severely	Extremely
24) Seizures impacted the quality of my life.	○	○	○	○	○
25) Short-term memory problems impacted the quality of my life.	○	○	○	○	○
26) Long-term memory problems impacted the quality of my life.	○	○	○	○	○
27) Problems in concentrating impacted the quality of my life.	○	○	○	○	○
28) Difficulties in making decisions impacted the quality of my life.	○	○	○	○	○
29) Problems with directions impacted the quality of my life (e.g. finding a parked car, losing my way in a familiar place).	○	○	○	○	○
30) Having less interest in activities I used to enjoy impacted the quality of my life.	○	○	○	○	○
31) Feelings of sadness impacted the quality of my life.	○	○	○	○	○
32) Feelings of anxiety impacted the quality of my life.	○	○	○	○	○
33) Being overly emotional impacted the quality of my life.	○	○	○	○	○
34) Being short-tempered or impatient impacted the quality of my life.	○	○	○	○	○
35) Weight gain impacted the quality of my life.	○	○	○	○	○

Activities of Daily Living (ADL) in the past 4 weeks

		Not at all / not applicable	Mildly	Moderately	Severely	Extremely
36)	My illness impacted my ability to do my job.	○	○	○	○	○
37)	My illness impacted my ability to do activities I used to enjoy.	○	○	○	○	○
38)	My illness impacted my ability to drive.	○	○	○	○	○
39)	My illness limited my ability to get around out of the house by myself.	○	○	○	○	○
40)	My illness limited my ability to do household chores by myself.	○	○	○	○	○
41)	My illness made me a burden to people around me.	○	○	○	○	○
