# Supplementary material for: Capturing what matters: Patient‐reported LGI1‐ANTibody encephalitis outcome RatiNg scale (LANTERN)
Source: Ann Clin Transl Neurol. 2025 Feb 25;12(4):821–31. doi: 10.1002/acn3.70006 (PMC12040509; doi:10.1002/acn3.70006)
Supplement: Supplementary file 9 — Data S1. [file ACN3-12-821-s003.docx]

**Supplemental materials – Glossary**

**Supplemental methods**

- - **Project proposal to develop a Patient-Reported Outcome Measure (PROM) in Autoimmune Encephalitis.**
    - This proposal was presented to participants of our study planning focus group
  - **Stage 1. Item generation interviews**
  - **Topic Guide for Qualitative Narrative PROM Interview (Stage 1)**
    - Used as a guide for interviewers in stage 1
  - **Topic Guide for cognitive debriefing**
    - Used as a guide for interviewers in stage 2

**Supplemental tables**

- - **1: Illustrative quotes**
  - **2: Summary statistics for standardised questionnaires**

**Supplemental files:**

- **Supplemental File 1 - Item Tracking Matrix Stage 1 (Formative interviews)**
- **Supplemental File 2 - Item Tracking Matrix Stage 2 (Cognitive Debriefing)**
- **Supplemental File 3a - Item-Tracking Matrix-Stage 3 (symptom burden)**
- **Supplemental File 3b - Item-Tracking Matrix-Stage 3 (ADL)**

**Supplemental questionnaires:**

- **Supplemental Questionnaire 1 - demographics and clinical info**
- **Supplemental Questionnaire 2- LANTERN_psychometric_survey**
- **Supplemental Questionnaire 3 - LANTERN_relatives**
- **Supplemental Questionnaire 4- LANTERN_final**

**Supplemental methods**

**Project proposal to develop a Patient-Reported Outcome Measure (PROM) in Autoimmune Encephalitis**

You are invited to take part in a focus group to give your opinion on, evaluate and refine a project being planned by the Oxford Autoimmune Neurology Group.

The aim of this project is to develop a patient reported outcome measure (PROM) for use in autoimmune encephalitis.

At this early stage, you are not being asked to participate in this study, though you may be asked in future. For now we are looking for opinions on our project design, to ensure we are best serving the interests of people with autoimmune encephalitis and their family members.

**What is a PROM?**

A PROM is a tool, usually a questionnaire, which is used to measure symptoms reported by patients, rather than by their doctors. This allows us to measure the symptoms of a condition that are most important to patients, and have the greatest impact on their quality of life.

PROMs can be used in research, including clinical trials and our clinical practice, to measure how effective a treatment is and to monitor an individual`s progress.

We at the Oxford Autoimmune Neurology Group are looking for help from people who have a history of autoimmune encephalitis, and from their caregivers.

**Why are PROMs important in autoimmune encephalitis?**

In our experience, many people with autoimmune encephalitis report symptoms such as fatigue and memory problems long after they have recovered form their initial illness. However, there are currently no PROMs designed to measure these symptoms in autoimmune encephalitis. The tools we currently use are typically completed by healthcare professionals.

We believe that future research in autoimmune encephalitis should use a PROM to measure these sort of long-term symptoms, so that we can focus on improving the symptoms most troublesome to patients.

**How can you help?**

We would like your feedback on the project we are planning. We want to develop a PROM for patients with autoimmune encephalitis. This would involve:

- Recruitment of people with autoimmune encephalitis that want to participate in our study
- 10-20 participants will have individual interviews to explore the aspects of their illness they find most troublesome. This interview will take between 1 and 2 hours.
- The interviews will be video-recorded and analysed afterwards
- On the basis of these interviews we will draft  a PROM questionnaire.
- The first version of the questionnaire may be presented to another group of participants (10-20 patients) and changed according to their input.
- The final questionnaire will be tested on 50-100 participants.

In future, we hope this PROM will be used in studies and clinical trials, aimed at improving the lives of people with autoimmune encephalitis.

**Thank you very much for considering taking part in helping to develop this project.**

**Stage 1. Item generation interviews**

To gather more detailed personal narratives than in focus groups, semi-structured interviews were conducted with 18 primary-participants (i.e. who had experienced LGI1-Ab-E). Interviews were face-to-face by one or two clinically experienced neurologists (BW, MJK), either alone or with a relative. These interviews documented all symptoms experienced since onset of illness, based on a semi-structured topic guide (see below; ).

Based on Consensus-based Standards for the selection of health Measurement INstruments (COSMIN) guidelines on PROM content validity,18 a fixed sample-size was not predetermined at the item generation stage. Instead, interviews were conducted until: 1) data saturation was reached – agreement between interviewers that no new relevant themes were being elicited, and 2) the characteristic 2:1 male:female ratio of LGI1-Ab-E was reached. Interviews were audio-recorded, transcribed verbatim, and underwent thematic analyses with a combination of inductive and deductive approach, generating domains and themes. Thematic analysis is a widely used qualitative analysis method which utilises codes to build themes in identifying repeating patterns.21, 22 All transcripts were reviewed and coded by at least one neurologist and 50% were independently coded by a second neurologist, for quality assurance. Both reviewers then reached consensus on the themes identified. These themes were discussed and verified with two other experienced neurologists (CS, SRI) to confirm clinical relevance.

These themes helped generate a comprehensive list of potential symptoms to include in LANTERN. Symptoms were included if identified in more than one interview and felt by authors to be clinically pertinent. Those mentioned only once were excluded to ensure relevance and minimise respondent burden (supplemental file 1 details items and rationale for including/removing).

**Topic Guide for Qualitative Narrative PROM Interview (Stage 1)**

**Documents given to patients prior to meeting via email**

- Invitation and information about study
- Questionnaires to fill out

**Before the Interview**

- Interviewer introduces themself
- Background information:

*“The aim is to develop a new questionnaire for patients with LGI1 antibody encephalitis. We will ask you in this interview about the symptoms of the disease that were the most troublesome to you. We are trying to identify those topics to then later design this new questionnaire. This is supposed to reflect the actual outcome of the patients in a better and more accurate way, than current measurements (blood test, MRI, clinical scores) do.”*

Important points

- We will video-record this interview and transcribe our conversation to be able to analyse it afterwards.
- Everything you say is confidential.
- The interview will take about 60 minutes, we can take breaks whenever you like.
- You can attend the interview alone, or with someone else present. We will ask both of your for your input, one after the other.
- There are no right or wrong answers, your opinion is important to us.
- Afterwards we will ask you to go through your clinical history together and do some memory tests to complete background information on your disease status
- Do you have any questions about this?

**The Interview**

Open ended questions for narrative PROM Interview

1) Describe all the symptoms that you associate with your disease!

2) In what way do you think you are different now compared to before your illness!

3) What aspects of your daily life have been affected by your illness?

Examples, if needed:

- activities impacted?

- how does it affect work?

- How does it affect enjoyable hobbies?

4) Can you think of anything in your life that has not been affected by your disease?

5) What do you think your partner/family member would say has changed since your illness?

6) What symptoms / affected activities do you feel your physicians may not routinely ask about or do not know about?

7) Any further problems you can think of?

Qs for partner:

Is there anything you would like to add?

Are they any other things you have noticed since his/her illness that have not been mentioned?

Prompting:

I will ask you about specific symptoms now (if you have not mentioned them already), and you can tell me, if these were relevant to you.

How is your sleep?

Do you feel Fatigue?

Did you notice an increased emotionality?

How is your memory?

Did you have seizures?

Did you have pain?

How is your mood?

**Stage 2. Initial item selection**

The purpose of these cognitive debriefings was to evaluate content validity in terms of comprehensiveness (ensuring all important ideas are covered), comprehension (ensuring instructions, items, and response options are understood as intended), and relevance (ensuring items, response options, and recall period are appropriate).^18^ Participants offered input on the 47 draft items via in-person or virtual interviews. Questions were grouped according to qualitative theme. Interviewers recorded comprehensive written notes. Participants read through each section of the questionnaire (including instructions) and provided thoughts on content (including instructions, response options, recall period, ordering, wording and types of questions, and whether any items were missing). See interviewer topic guide (Supplemental materials).

Within the third cognitive debriefing round, a stakeholder meeting involving the study group evaluated the comprehensiveness, comprehension and relevance in the same way as participants. Changes derived from this meeting were applied and the revised questionnaire shown to two further participants with LGI1-Ab-E and two relatives.

At the end of an interview round, the two interviewers assessed the quality of the questions under several established domains, based on participant responses:^16^ Does it reflect quality-of-life?; Ease-of-completion; Not value laden; Avoids interpersonal and intertemporal comparison; Psychometric performance; Suitability for evaluation; Comprehensibility; Relevance; Comprehensiveness; Avoids redundancy/overlap. Finally, interviewers reached consensus on whether a question should be retained, altered or removed, and whether additional questions should be added (Supplemental file 2).

**Topic Guide for cognitive debriefing**

**Documents given to patients prior to meeting via email**

- Invitation letter with information about study
- Draft PROM
- RedCap Link with other existing Questionnaires to fill out for external validation later on

**Before the interview**

- Interviewer introduces him/herself
- Background information:

*“This study is being carried out by the University of Oxford. The aim is to develop a new questionnaire for assessing outcome and quality of life in patients with LGI1 antibody encephalitis. We have spoken to a number patients and used that information to produce some draft questions for that questionnaire.*

*With your help, we would like to go through these draft questions in this study, to see if they make sense to you, to see if you consider them relevant, to see which questions you prefer, and to see if there is anything important to you that has not been included. We are asking patients, their family members/spouses, and some doctors to help us with this.”*

- Important points:
  - We may record this interview, and we will take notes to improve the questionnaire
  - Everything you say is confidential
  - The interview will take up to 90 minutes, we can take a break whenever you like.
  - You can attend the interview alone or with someone else, but for this part we want to focus on your opinion
  - There are no right or wrong answers, your opinion is important to us
  - Afterwards we will ask you to go through your clinical history together and do some memory tests to complete background information on your disease status
  - Do you have any questions about this?

**During the interview**

- Instructions: Can you please read the instructions. In your own words, can you tell me what the instructions are asking you to do? Do you understand what to do in completing the questionnaire? Are there any words or phrases you would change to improve the instructions? We have a left and a right colum of questionnaires. Do you understand those ?
- Response options: Consider the responses you can give to the questions: “less than every month/Never”, “Every Month”, “Every week”, “Every day”, “Multiple times per day” – do they make sense to you? What does response [X] mean to you? Can you please describe the difference between them? Would you ever choose option [X], why or why not? Can you describe a situation where you would choose [X]? Separate options would be: “Never”, “seldom/Rarely”, “sometimes”, “frequently/Often”, “always” – or
- Recall: The questions ask about the last 4 weeks. Describe your experiences with symptoms of LGI antibody disease over the past 4 weeks. Is this a relevant timeframe to ask you about your symptoms and their impacts? Can you remember over the last 4 weeks? Would you prefer a different time period?For some questions we want you to think about different ways we could ask the same question
- Individual questions:
  - Using your own words, how would you explain what this question means?
  - What does [item X] mean to you?
  - What do you think of that question? Does it make sense?
  - Is it upsetting or frustrating?

Are any words difficult to understand? Are there any words you would change? Is there a better way to ask it?

- - Is the question relevant or redundant?
  - Are you able to answer it, using the options provided?
  - In case of similar/grouped questions: Do you think they are asking the same thing? Which one do you prefer? Why?
- Comprehensiveness: Are there any other important symptoms or impacts of symptoms on your daily life of LGI1 antibody disease, that you think the questionnaire is missing?
- Format: Do you have a preferred order to ask you the questions?
- Do you find it helpful to have questions on both frequency and impact (left and right columns). Would you prefer one or the other? Or a mix of the two?
- Past or present tense?
- Frequency: Do you prefer examples (daily, etc.)
- “+/- people around you” – Is this a helpful addition.Or rather extra questionnaire to fill out for wife?

**Close**

*“That’s the end of the interview. Thanks for your contribution it is very much appreciated. Do you have any questions? Thank you for your time. “*
