# Supplementary material for: Capturing what matters: Patient‐reported LGI1‐ANTibody encephalitis outcome RatiNg scale (LANTERN)
Source: Ann Clin Transl Neurol. 2025 Feb 25;12(4):821–31. doi: 10.1002/acn3.70006 (PMC12040509; doi:10.1002/acn3.70006)
Supplement: Supplementary file 10 — Table S1. [file ACN3-12-821-s008.docx]

**Supplemental table 1. Illustrative quotes** from participants highlighting symptoms and ADLs included in the final questionnaire.

| Domain | Item | Quote |
| --- | --- | --- |
| Physical | Weakness | “I feel as if I have lost 15% of my muscles and noticed a loss of strength.”  “I felt weak and displaced.” |
|  | Needing assistance | “I mainly rely on my wife, hardly go outside on my own, which makes me feel grateful and guilty at the same time.” |
|  |  |  |
|  | Physical fatigue | “I am tired within an hour of gardening, which was not the case before.” |
|  | Mental fatigue | “I will fall asleep after reading 3 pages of a book.”  “I cannot drive more than one hour, because of sleepiness.”  “I felt sleepy during the day, and have to sleep at work.” |
| Sleep | Trouble sleeping | “I had a very interrupted sleep, sleeping in “bits” of 2 hours.”  “I could only sleep 3 hours a night.” |
|  | Sleeping excessively | “Early in the illness, I slept 16 hours a day.” |
| Cognition | Concentration | “I feel like I have a fuzzy brain, it`s hard to concentrate.” |
|  | Focal seizures | “I noticed a sweeping through the body, like being frozen.”  “It felt like watching a scary movie, when you get pins and needles.”  “I had “out of body” or “our of space” sensations.”  “It started with those wobbly attacks, how I called them, a wave of sensation through my body and often the coffee would jump out of my hand.”  “It last seconds and in the acute phase, it came every 3 minutes.” |
|  | Generalized seizures | “It began with a longer episode of confusion, my wife thought, I was having a stroke, called the ambulance, when I had a witnessed generalized tonic-clonic seizure.” |
|  | Weight gain | “I gained weight due to the steroids.”  “For me the weight-gain due to the steroids had the biggest impact on my quality of life. I did not dare to attend video calls because of the round steroid face.” |
|  | Short-term memory | “I have a poor short-term memory. It feels like the movie, “Groundhog Day”. Every day is a new day. There is an element of this.”  “I write down everything after I have had a conversation with a person, so next time I meet that person, I can check with my notes first.”  “I kept forgetting if I had brushed my teeth already, and would touch the toothbrush to feel if it is still wet.” |
|  | Directions | “I got lost in a very small train station, and had a panic attack”  “I would get lost on a straight street.”  “I could not find my way back to the car.” |
|  | Decision-making | “I wanted to do something, but could not decide what to do.” |
| Emotional/  behavioural | Emotionality | “I cry more easily than before”  “I mirror peoples’ emotions”  “I noticed I cry more easily after watching things on TV”  “When I had encephalitis and watched an Oxfam advert about children starving in Africa, I would start crying and couldn`t stop for at least 5 minutes. Now, when I watch an Oxfam advert, I still get very emotional, but don’t cry.”  “When I heard an animal had died, I cried, even though I didn`t know the animal.”  “I used to be able to control my emotions very well, and now I can`t control them anymore.”  “I am crying at movies, which I never used to do.” |
|  | Long-term memory | “I first noticed that I do not remember major events of my life, that had happened in the past. Now I realise that I am still missing the last 9-10 years of my life completely.” |
|  | Low mood | “I felt depressed and only wanted to be left alone.”  “It felt depressing to fail again, and not being able to remember.”  “I was depressed, only sat on a sofa and watched TV, did not want to go outside.” |
|  | Anxiety | “I was anxious and fearful of doing something wrong.”  “I was taking everything personally.” |
|  | Short temper | “I was short-tempered, impatient.”  “I had mood swings and was more irritable and frustrated.”  According to relative: “He sometimes blows off the top of the hat out of nothing. If he does something, you cannot interrupt him”.  “I am less tolerant of dogs barking, more sensitive to noise.” |
|  | Interest in previously enjoyed activities | “I had less interest, could not be bothered to do things.” |
| ADL | Job | “I could not do my prior job, mainly due to memory problems.”  “I cannot work, because of fatigue and memory issues.” |
|  | Previously enjoyed activities | “Enjoyable hobbies, like watching football is more difficult, because I do not remember the players, like I used to.”  “I cannot read a novel anymore, because I cannot remember the context, and not follow the story. Therefore, I prefer history books instead.” |
|  | Driving | “I cannot drive, and therefore cannot work anymore, which has been a huge impact on my quality of life. “ |
|  | Mobility | “I was unsteady on my feet.” |
|  | Being a burden on others | “I felt like I was a burden to everyone.” |

**Supplemental table 2. Summary statistics for standardised questionnaires.** All 66 participants were asked to complete standardised questionnaires covering the different domains of the PROM. Where applicable, clinically recognised cut-offs are applied to identify those impaired.

| **Measure** | **Cut-off for impairment** | **Median (range)** | **N impaired** |
| --- | --- | --- | --- |
| **mRS** | **> 2** | 2 (0, 3) | 10/61 (16.4%) |
| **CASE** | **N/A** | 2 (0, 5) | N/A |
| **EQ5D-VAS** | **N/A** | 75 (11, 100) | N/A |
| **MFIS** | **> 38** | 30 (0, 84) | 27/66 (40.9%) |
| **HADS-A** | **>/= 7** | 4 (0, 16) | 20/65 (30.8%) |
| **HADS-D** | **>/= 8** | 5 (0, 14) | 24/65 (36.9%) |
| **NQ-EBD** | **> 60** | 49.4 (29.5, 71.9) | 7/62 (11.3%) |
| **NQ-Cog** | **< 40** | 48.3 (22.6, 59) | 10/65 (15.4%) |
